# Factors associated with perinatal and neonatal deaths in Sao Tome & Principe: a prospective cohort study

**DOI:** 10.3389/fped.2024.1335926

**Published:** 2024-02-16

**Authors:** Alexandra Vasconcelos, Swasilanne Sousa, Nelson Bandeira, Marta Alves, Ana Luísa Papoila, Filomena Pereira, Maria Céu Machado

**Affiliations:** ^1^ Unidade de Clínica Tropical—Global Health and Tropical Medicine (GHTM), Instituto de Higiene e Medicina Tropical (IHMT), Universidade NOVA de Lisboa, Lisboa, Portugal; ^2^ Department of Pediatrics, Hospital Dr. Ayres de Menezes, São Tomé, Sao Tome and Principe; ^3^ Department of Obstetrics & Gynecology, Hospital Dr. Ayres de Menezes, São Tomé, Sao Tome and Principe; ^4^ CEAUL, NOVA Medical School/Faculdade de Ciências Médicas, Universidade NOVA de Lisboa, Lisboa, Portugal; ^5^ Faculdade de Medicina de Lisboa, Universidade de Lisboa, Lisboa, Portugal

**Keywords:** neonatal mortality, perinatal mortality, stillbirth, neonatal death, Sao Tome & Principe

## Abstract

**Background:**

Neonatal mortality reduction is a global goal, but its factors are seldom studied in most resource-constrained settings. This is the first study conducted to identify the factors affecting perinatal and neonatal deaths in Sao Tome & Principe (STP), the smallest Central Africa country.

**Methods:**

Institution-based prospective cohort study conducted at Hospital Dr. Ayres Menezes. Maternal-neonate dyads enrolled were followed up after the 28th day of life (*n* = 194) for identification of neonatal death-outcome (*n* = 22) and alive-outcome groups (*n* = 172). Data were collected from pregnancy cards, hospital records and face-to-face interviews. After the 28th day of birth, a phone call was made to evaluate the newborn's health status. Crude odds ratios and corresponding 95% confidence intervals were obtained. A *p* value <0.05 was considered statistically significant.

**Results:**

The mean gestational age of the death-outcome and alive-outcome groups was 36 (SD = 4.8) and 39 (SD = 1.4) weeks, respectively. Death-outcome group (*n* = 22) included sixteen stillbirths, four early and two late neonatal deaths. High-risk pregnancy score [cOR 2.91, 95% CI: 1.18–7.22], meconium-stained fluid [cOR 4.38, 95% CI: 1.74–10.98], prolonged rupture of membranes [cOR 4.84, 95% CI: 1.47–15.93], transfer from another unit [cOR 6.08, 95% CI:1.95–18.90], and instrumental vaginal delivery [cOR 8.90, 95% CI: 1.68–47.21], were factors significantly associated with deaths. The odds of experiencing death were higher for newborns with infectious risk, IUGR, resuscitation maneuvers, fetal distress at birth, birth asphyxia, and unit care admission. Female newborn [cOR 0.37, 95% CI: 0.14–1.00] and birth weight of more than 2,500 g [cOR 0.017, 95% CI: 0.002–0.162] were found to be protective factors.

**Conclusion:**

Factors such as having a high-risk pregnancy score, meconium-stained amniotic fluid, prolonged rupture of membranes, being transferred from another unit, and an instrumental-assisted vaginal delivery increased 4– to 9–fold the risk of stillbirth and neonatal deaths. Thus, avoiding delays in prompt intrapartum care is a key strategy to implement in Sao Tome & Principe.

## Introduction

The first 28 days of life—the neonatal period—are the most vulnerable days for a child's survival (1, 2). Even nowadays, a significant number of babies die before birth, never having the chance to take their first breath (1, 2). The terminology used depends on the time of death. Perinatal mortality includes stillbirths and early neonatal deaths (ENND), indicating the death of a live newborn before the age of seven completed days (1). Late neonatal deaths (LNNDs) are those that occur after 7 days to 28 completed days of birth (2).

Globally, perinatal mortality accounts for three-fourths of deaths during the neonatal period (3, 4). More than half of the cases of stillbirths occur when pregnant women are in labor, and these deaths are directly related to the lack of skilled care at this critical time (5, 6). On the other hand, the largest contributors to neonatal mortality (ENND plus LNND) are complications of preterm birth, birth asphyxia, infection, and congenital malformations although they can differ depending on the country context (7).

Only in the last two decades, mainly after Lawn et al. published article titled “4 million neonatal deaths: When? Where? Why?” (8), attention started to be given to the neonatal period in developing countries although stillbirths are still invisible and missing from the Sustainable Development Goals (SDG) agenda (5, 9, 10). It urges to highlight that stillbirths and newborn deaths account for twice as many deaths as malaria and human immunodeficiency virus (HIV) infection combined but have received much less awareness and funding in these resource-constrained countries (8, 9).

Understanding the causes of stillbirths (fetal deaths) is also complex, as there are many promoting and interacting factors (11). In most low –to medium—income countries (LMICs), it is difficult to determine the exact reason for the stillbirth; therefore, the cause of death is often classified as “unexplained” (5). The definition of stillbirth used in this study was the WHO/ICD (for international comparison and reporting) as a baby born without any signs of life at or after 28 weeks of gestation or at least 1,000 g in birth weight (9, 12). Intrapartum stillbirth was defined as a dead-born fetus where intrauterine death occurred after the onset of labor and before birth (fresh stillbirth) (9). Antepartum stillbirth is a dead born fetus where intrauterine death occurs before the onset of labor (macerated stillbirth) (9). Researchers report that different risk factors for fetal death, such as maternal factors (advanced maternal age, high pre-pregnancy body mass index, smoking, low socioeconomic status), obstetric history (grand multiparity, previous stillbirth), antepartum factors (fewer than four antenatal visits, fetal growth restriction, maternal anemia, maternal fever and infections, antepartum hemorrhage, hypertension), and intrapartum factors (preterm birth, extremes of neonatal birth weight, cesarean delivery, operative vaginal delivery, and assisted breech delivery), are all factors that have been reported in various studies as causes of stillbirths (6, 11, 13–16).

Sao Tome & Principe (STP) is a LMIC with low HIV/AIDS prevalence and a malaria pre-elimination phase (17, 18). On the other hand, perinatal and neonatal mortality rates are considered a public health problem, given that neonatal deaths account for approximately 43% of all under5 deaths (19). At the time this study was initiated in 2016, there was an annual rate of 22 stillbirths and 22 newborn deaths per 1,000 livebirths (19). There is an established antenatal care (ANC) service in STP, with a 98% rate of women seen at least once by a skilled health provider during pregnancy and a 95.4% rate of deliveries occurring in health units (18). There is no health insurance policy in the country or any private maternity units. In STP, there are three to four obstetricians, one anesthesiologist and one to two general doctors who provide care to the neonates (20). There are no neonatologists in the country. The midwives in the labor ward are responsible for the initial resuscitation of normal deliveries, and doctors from the pediatric department are called to the labor ward to attend babies in distress.

The HAM maternity unit has a facility-based clinical care unit for ill newborn babies, but there is no neonatal or child intensive care unit in the country.

We are aware that the concept of knowing what works in terms of reducing perinatal and neonatal mortality is complicated by a huge diversity of country contexts and of determinants of maternal and neonatal health (21–24). However, according to Lawn and other authors, identifying and addressing avoidable causes of neonatal death is possible even in poorly functioning health systems, justifying our current study (8, 9). Answering—why, when, and where—newborns die in Sao Tome & Principe will enable the design of appropriate planning to prevent this major public health problem since, for appropriate prevention of fetal and newborn mortality, data pertaining to its determinants are important.

To the best of our knowledge, this is the first study to assess perinatal and neonatal deaths in STP, and it was undertaken within the context of a broader project on neonatal adverse birth outcomes (ABOs) and other maternal problems in this LMIC (25–31).

Therefore, we conducted a prospective cohort study to identify the most important factors associated with death among newborns delivered at HAM.

## Materials and methods

### Study design and period

An institution-based prospective cohort study was conducted at Hospital Dr. Ayres de Menezes (HAM) for mother-neonate dyads followed up until the 28th day after delivery (neonatal period). Recruitment of participants (mother-newborn dyad) occurred from July 2016 to November 2018.

### Setting

The archipelago of Sao Tome & Principe is one of the smallest sub-Saharan African (SSA) countries, with approximately 200.000 inhabitants and a total land surface of approximately 1,001 km^2^ on two islands (Sao Tome and a smaller island named Principe) (17, 18). As a tertiary healthcare facility, HAM receives the most complicated cases from facilities in lower levels of care. The Neonatal Care Unit (NCU) receives high-risk babies delivered within the institution and referrals from other health facilities or from home with a total capacity to admit six babies. This unit, like others in SSA settings, is basic and able to manage simple neonatal complications such as hypothermia, feeding problems and sepsis suspicion. Although NCU was rebuilt in 2016, there is still a lack of continuous positive airway pressure therapy, surfactant therapy and enteric feeding for assisting sick babies.

### Study population and follow-up

All mother-neonate dyads admitted to the HAM maternity unit for childbirth constituted the source population whereas the study populations were selected neonates delivered in the HAM maternity unit during the study period. During the study period, 4,540 deliveries were recorded, corresponding to 450 cesarean deliveries and 3,740 normal vaginal births.

The inclusion criteria for participants were as follows: (1) all neonates delivered at HAM with a gestational age of 28 weeks or more and (2) newborns who were born outside the hospital but were later admitted at HAM within the first 12 h of life. A total of 535 newborns were initially enrolled.

The exclusion criteria included the following: (1) all neonates delivered at HAM with a gestational age of less than 28 weeks, (2) newborns whose mothers had no antenatal pregnancy card, (3) newborns whose mothers had cognitive impairment. The newborn was also excluded if his health status was unknown at his twenty-eight days of life.

**Figure 1:**
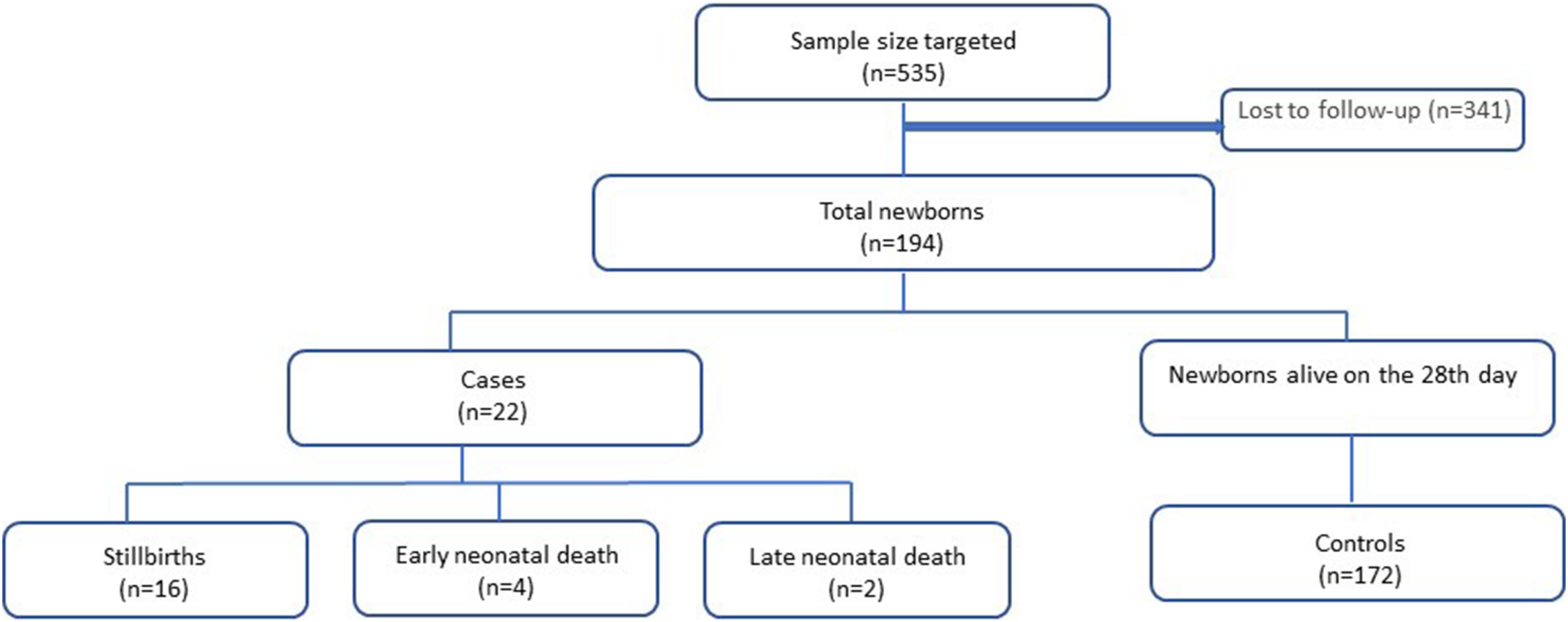
Flowchart of participation in the study.

Consenting participants in the sample were followed up (mother-newborn dyad) throughout their stays until hospital discharge. The survival status of neonates after discharge was ascertained by making a follow-up mobile phone call at the end of the neonatal period. Those who could not be reached by phone after four attempts in different weeks were taken as nonrespondents, and the mother-newborn dyad was excluded. A flowchart of participation in the study is shown in Figure 1.

### Sampling method

This is a substudy undertaken to estimate adverse birth outcomes (ABOs) and related risk factors in the country.

The sample size initially calculated for the broader project, using Raosoft® (http://www.raosoft.com/samplesize.html), was based on the formula for sample size and margin of error from the software, reaching the minimum sample size of S  =  355, with 355 (95%) and 579 (99%) interval confidence. The original study included 537 mother-neonate dyads enrolled based on the following assumptions: two-sided 95% confidence level, power of 80% to detect an odds ratio of at least two for ABO. Since the sample size was not calculated for present outcomes, to assess perinatal and neonatal deaths, a power analysis was performed, varying from 77% to 87% for outcomes such as gestational age (GA < 37) for this study. In this study, 194 mother-neonate dyads were included. Participants were selected through random sampling. Each morning, from the pile of motherś medical folders, every second interval folder was selected and then carried on requesting contentment for enrollment. To guarantee a sample with few biases and effects by means of confounding variables, the study was conducted in different months (two weeks every two other months), avoiding seasonal interference (rain season and malaria period). Women were interviewed only after the delivery, although the invitation and consent were obtained during her admission to the maternity unit before birth (live birth or stillbirth).

### Selection of groups: death and alive neonatal outcome

A total of 194 mother-neonate dyads were included and were divided into two groups according to the newborn status (alive vs. dead) at the end of the neonatal period. Newborns who died before the 28th day of life (death-outcome group with stillbirths included; *n* = 22) were compared with newborns alive at the 28th day of life (alive-outcome group; *n* = 172) concerning maternal, antepartum, and intrapartum characteristics.

### Study variables

The dependent variables for the study were death (stillbirth and neonatal—early and late-death) and live newborns.

For neonates who died at home after discharge, probable causes of death were assigned by the principal investigator (pediatrician) using the International Statistical Classification of Diseases and Related Health Problems ICD-11 coding. For neonates who died in the HAM, the cause of death was assigned by physicians, who work in the hospital, and confirmed by the principal investigator.

The independent variables tested in this study included factors grouped into five categories: (1) newborns' maternal sociodemographic factors (age, educational status, occupation, marital status, partner's education, and residence); (2) preconception factors (previous contraceptive utilization), plus current obstetric condition (gravidity, parity, previous abortion and stillbirth, previous cesarean section and preceding birth interval); (3) ANC service (number of visits, gestational age at first ANC visit, obstetric ultrasound, number of fetuses in the ultrasound) plus antepartum factors as positive ANC screenings [high-pregnancy risk score, maternal anemia as hemoglobin concentration <11 g/dl, bacteriuria, hyperglycemia, Rh incompatibility, intestinal parasitic infection (IPI), malaria, HIV, syphilis, hepatitis B virus and sickle cell]; (4) health facility-related factors (being transferred from another unit, who assisted the delivery and partograph use) plus intrapartum factors as mode of delivery and complications (fetal malpresentation (32), umbilical cord complication (33), prolonged rupture of membranes (PROM) (34, 35), meconium-stained amniotic fluid (36), postpartum hemorrhage as bleeding >500 ml, preeclampsia defined as hypertension ≥140/90 mmHg and proteinuria in dipsticks in women who were normotensive at ANC, and obstructed labor (37); (5) newborns characteristics as (gestational age, sex, birth weight) and complications [intrauterine growth restriction (IUGR), congenital anomalies, infectious risk, neonatal resuscitation, fetal distress at birth, birth asphyxia and admission at NCU] (38–40).

### Data collection

Data on antepartum, intrapartum, and postpartum characteristics of participants were gathered and collected from ANC pregnancy cards, obstetric maternal and newborn clinical records. For antepartum data, relevant details of the perinatal history and antenatal period were collected systematically from the ANC pregnancy card. Intrapartum data were collected from labor follow-up sheets, delivery summaries and maternal medical records. Postpartum data were abstracted from newborn birth charts and/or newborn medical records if admitted to the NCU.

Maternal sociodemographic characteristics were supplemented with a structured administered questionnaire through a face-to-face interview of the mothers 12–24 h after delivery, similar to other studies from LMICs (41).

### Data quality alive-outcome group

The questionnaires were administered in Portuguese, the country national language. The questionnaire was pretested at HAM one month before data collection in 23 mothers, and modification was made based on the pretest result, mainly adjusting terminology for more culturally friendly terms. Womeńs consent to participate in the study was obtained at the time of admission at HAM, but the interview was held after a woman was stabilized and ready to be discharged. Continuous follow-up and supervision of data collection were made by the supervisors. The collected data were checked daily for completeness. The principal investigator (pediatrician) executed and was responsible for all main activities as follows: (1) obtaining consent and enrollment of the participants, (2) data collection from antenatal cards plus maternal clinical and newborns' records, (3) newborns' clinical observation (for diagnosis confirmation), (4) face-to-face interviews, (5) administering all phone interviews and (6) data collection entry into the database.

### Data management

The anonymity and safety of the participants were ensured. Data were secured in a confidential and private location. Participants were referred to by identification numbers, and the informed consent forms were kept separate from the questionnaires. Both could only be linked by a coding sheet available only to the principal investigator.

### Data analysis

Data were entered into the QuickTapSurvey app (©2010–2021 Formstack), and the dataset was exported to Excel for cleaning and further analysis using the Statistical Package for the Social Sciences for Windows, version 25.0 (IBM Corp. Released 2017. IBM SPSS Statistics for Windows, Version 25.0. Armonk, NY: IBM Corp.) and Stata 15.0 (StataCorp. 2017. Stata Statistical Software: Release 15. College Station, TX: StataCorp LLC). All data were checked for completeness and accuracy by the principal investigator and a qualified biostatistician.

Descriptive statistics, namely, frequencies and percentages were estimated. For binary variables, crude odds ratios estimates with 95% confidence intervals (95% CI) were obtained from corresponding contingency tables, and for categorical variables with more than two categories, exact logistic regression was used. In this study, the neonatal death-outcome group was coded as 1, and the neonatal alive-outcome group was coded as 0. The proportion of missing data ranged from 0.8 to 10% across variables, and missing values higher than 10% were described in the analysis. A level of significance *α* = 0.05 was considered.

## Results

A total of 194 newborns were followed up during their first 28 days of life. In this study, the newborn's mean gestational age (GA) was 38.86 weeks with a standard deviation (SD) of 2.26 (minimum 28–maximum 42 weeks). The death-outcome group (*n* = 22) had a mean GA and birth weight of 36 (SD = 4.83) and 2,515 (SD = 997) g, respectively, while their counterparts (172 in the alive-outcome group) had 39 (SD = 1.41) weeks and 3,209 (SD = 507) g, respectively. The mean maternal age was 27.14 years, with a SD of 6.86 (minimum 15–maximum 43) years old. The mean maternal age for the death-outcome group and alive-outcome group was 30.73 (SD = 7.45) and 26.68 (SD = 6.66) years, respectively.

The death-outcome group under-study included 16 stillbirths (72.7%), four (18%) ENND and two (9%) LNND. Stillbirths were 69% intrapartum stillbirths (fresh stillbirths), and 31% were antepartum stillbirths (macerated stillbirths). Stillbirth characteristics are further described in Supplementary Additional File S1, and the early and late neonatal deaths (ENND and LNND) are described in Supplementary Additional File S2.

The maternal characteristics as well as antepartum, intrapartum, and postpartum factors for the total of the participants and for the death-outcome group vs. the alive-outcome group are described in Tables 1, 2.

**Table 1:** Univariable analysis of perinatal and neonatal deaths among newborns admitted at HAM, Sao Tome & Principe (*n* = 194; death-outcome group = 22 and alive-outcome group = 172).

Variables	Categories	Total	Death-outcome group	Alive-outcome group	cOR	(95%) CI	*p* value
		*n* = 194	*n* = 22	*n* = 172			
			*n* (%)	*n* (%)			
Sociodemographic characteristics							
Motheŕs age	14–19	28 (14.4)	3 (13.6)	25 (14.5)	1		
	20–34	131 (67.5)	11 (50.0)	120 (69.8)	0.765	0.183–4.578	0.925
	≥35	35 (18)	8 (36.4)	27 (15.7)	2.436	0.510–15.842	0.355
Motheŕs education	None + primary	106 (54.6)	19 (86.4)	87 (50.6)	1		
	Secondary	88 (45.4)	3 (13.6)	85 (45.4)	0.162	0.046–0.566	**0** **.** **002**
Motheŕs occupation	Unemployed	139 (72.0)	16 (72.7)	123 (71.9)	1		
	Employed	54 (28)	6 (27.3)	48 (28.1)	0.961	0.355–2.601	0.937
Marital status	Union/married	124 (63.9)	14 (63.6)	110 (64)	0.986	0.392–2.482	0.977
	Single	70 (36.1)	8 (36.4)	62 (36)	1		
Babýs father education	None + primary	63 (41.7)	9 (81.8)	54 (38.6)	1		
	Secondary	88 (58.3)	2 (18.2)	86 (61.4)	0.140	0.029–0.67	**0** **.** **006**
Residence^a^	Urban	92 (48.2)	9 (45)	83 (48.5)	0.867	0.342–2.200	0.817
	Rural	99 (51.8)	11 (55)	88 (51.5)	1		
Preconceptional							
Contraception previous use	Yes	38 (24.8)	3 (20)	35 (25.4)	0.736	0.196–2.760	0.763
	No	115 (75.2)	12 (80)	103 (74.6)	1		
Obstetric history							
Gravidity	1	42 (21.6)	4 (18.2)	38 (22.1)	1		
	2–5	100 (51.5)	8 (36.4)	92 (53.5)	0.827	0.206–3.981	0.995
	≥5	52 (26.8)	10 (45.5)	42 (24.4)	2.243	0.585–10.628	0.306
Parity	0	53 (27.3)	5 (22.7)	48 (27.9)	1		
	1–4	121 (62.4)	12 (54.5)	109 (63.4)	1.057	0.324–4.045	1.000
	≥5	20 (10.3)	5 (22.7)	15 (8.7)	3.140	0.631–15.764	0.186
Previous abortion	Yes	59 (30.4)	6 (27.3)	53 (30.8)	0.842	0.312–2.272	0.734
	No	135 (69.6)	16 (72.7)	119 (69.2)	1		
Previous stillbirth	Yes	17 (8.8)	4 (18.2)	13 (7.6)	2.718	0.801–9.225	0.109
	No	177 (91.2)	18 (81.8)	159 (92.4)	1		
Poor birth spacing^b^	Yes	39 (20.1)	6 (27.3)	33 (19.2)	1.580	0.574–4.346	0.399
	No	155 (79.9)	16 (72.7)	139 (80.8)	1		
Antenatal care							
GA at first ANC visit	≤12	98 (60.5)	6 (27.3)	92 (53.5)	1		
	>12	58 (35.8)	9 (40.9)	55 (32)	2.509	0.847–7.430	0.088
Number of ANC visits	1–4	19 (10.1)	2 (10)	17 (10.1)	1		
	5–7	84 (44.4)	16 (80)	68 (40.2)	1.988	0.402–19.493	0.609
	≥8	86 (45.5)	2 (10)	84 (49.7)	0.207	0.014–3.035	0.298
Obstetric ultrasound	0	73 (38.4)	7 (35)	66 (38.8)	1		
	1	87 (45.8)	12 (60)	75 (44.1)	1.505	0.511–4.792	0.570
	2	30 (15.8)	1 (5)	29 (17.1)	0.328	0.007–2.745	0.524
Twin pregnancy	Yes	14 (7.2)	0	14 (8.1)	-	-	0.375
	No	180 (92.8)	22 (100)	158 (91.9)	1		
Antenatal care screenings							
High-pregnancy risk^c^	Yes	70 (36.1)	13 (59.1)	57 (33.1)	2.914	1.176–7.220	**0** **.** **017**
	No	124 (63.9)	9 (40.9)	115 (66.9)	1		
Maternal disease during pregnancy^d^	Yes	123 (63.4)	11 (50)	112 (65.1)	0.624	0.220–1.809	0.448
	No	66 (34)	9 (40.9)	57 (33.1)	1		
	Missing	5 (2.6)	2 (9.1)	3 (1.7)	4.100	0.304–41.348	0.337
Maternal anaemia^e^	Yes	59 (30.4)	6 (27.3)	53 (30.8)	1.729	0.438–6.830	0.531
	No	98 (50.5)	6 (27.3)	92 (53.5)	1		
	Not tested	37 (19.1)	10 (45.5)	27 (15.7)	5.588	1.666–20.543	0.004
Bacteriuria	Yes	62 (32)	5 (22.7)	57 (33.1)	0.593	0.208–1.690	0.324
	No	132 (68)	17 (77.3)	115 (66.9)	1		
Hyperglycaemia^f^	Yes	8 (4.1)	1 (4.5)	7 (4.1)	1.122	0.132–9.578	1.000
	No	186 (95.9)	21 (95.5)	165 (95.9)	1		
Rh incompatibility	Yes	7 (3.6)	0	7 (4.1)	-	-	1.000
	No	187 (96.4)	22 (100)	165 (95.9)	1		
IPIs	Yes	98 (50.5)	8 (36.4)	90 (52.3)	0.521	0.208–1.305	0.159
	No	96 (49.5)	14 (63.6)	82 (47.7)	1		
Malaria	Yes	1 (0.5)	0	1 (0.6)	-	-	1.000
	No	193 (99.5)	22 (100)	171 (99.4)	1		
HIV	Yes	2 (1.0)	1 (5.0)	1 (0.6)	1		
	No	192 (99)	19 (95)	163 (99.4)	0.123	0.007–2.037	0.206
HsAg positive	Yes	6 (3.1)	2 (9.1)	4 (2.3)	1		
	No	113 (58.2)	10 (45.5)	103 (59.9)	0.194	0.032–1.195	0.112
Sickle cell positivity^g^	Yes	13 (6.8)	1 (4.5)	12 (7.0)	0.631	0.078–5.102	1.000
	No	181 (93.2)	21 (95.5)	160 (93)	1		
Health facility-related factors							
Baby delivered at HAM	Yes	188 (96.9)	21 (95.5)	167 (97.1)	1.590	0.177–14.275	0.519
	No	6 (3.1)	1 (4.5)	5 (2.9)	1		
Transferred from another unit*	Yes	16 (8.2)	6 (27.3)	10 (5.8)	6.075	1.953–18.900	**0** **.** **004**
	No	178 (91.8)	16 (72.7)	162 (94.2)	1		
Delivery assisted by	Obstetrician	36 (18.6)	5 (22.7)	31 (18)	1		
	Midwife	153 (78.9)	16 (72.7)	137 (79.7)	0.725	0.231–2.726	0.736
	Home labor	5 (2.6)	1 (4.5)	4 (2.3)	1.532	0.026–20.683	1.000
Partograph use	Yes	76 (39.2)	9 (40.9)	67 (39)	1.085	0.440–2.678	1.000
	No	118 (60.8)	13 (59.1)	105 (61)	1		
Fetal malpresentation^h^	Yes	2 (1)	0	2 (1.2)	-	-	1.000
	No	192 (99)	22 (100)	170 (98.8)			
PROM^i^	Yes	15 (8.1)	5 (23.8)	10 (6.1)	4.844	1.473–15.930	**0** **.** **016**
	No	171 (91.9)	16 (76.2)	155 (93.9)	1		
Pre/Eclampsia^j^	Yes	17 (8.8)	2 (9.1)	15 (8.7)	1.047	0.223–4.917	1.000
	No	177 (91.2)	20 (90.9)	157 (91.3)	1		
Obstructed labour^k^	Yes	22 (11.3)	4 (18.2)	18 (10.5)	1.901	0.579–6.239	0.286
	No	172 (88.7)	18 (81.8)	154 (89.5)	1		
Postpartum haemorrhage^l^	Yes	2 (1)	0	2 (1.2)	-	-	1.000
	No	192 (99)	22 (100)	170 (98.8)	1		
Normal Vaginal delivery	Yes	155 (79.9)	15 (68.2)	140 (81.4)	0.490	0.185–1.300	0.161
	No	39 (20.1)	7 (31.8)	32 (18.6)	1		
Caesarean section	Yes	33 (17)	4 (18.2)	29 (16.9)	1.096	0.345–3.477	0.772
	No	161 (83)	18 (81.8)	143 (83.1)	1		
Previous caesarean	Yes	10 (5.2)	2 (9.1)	8 (4.7)	2.050	0.407–10.333	0.316
	No	184 (94.8)	20 (90.9)	164 (95.3)	1		
Instrumental vaginal delivery^m^	Yes	6 (3.1)	3 (13.6)	3 (1.7)	8.895	1.676–47.208	**0** **.** **020**
	No	188 (96.9)	19 (86.4)	169 (98.3)	1		** **
Meconium-stained amniotic fluid	Yes	43 (22.2)	11 (50)	32 (18.6)	4.375	1.744–10.975	**0** **.** **002**
	No	151 (77.8)	11 (50)	140 (81.4)	1		
Umbilical cord complication	Yes	8 (4.1)	1 (4.5)	7 (4.1)	1.122	0.132–9.578	1.000
	No	186 (95.9)	21 (95.5)	165 (95.9)	1		
Newborn's characteristics							
Gestational Age^n^	28–31	3 (1.5)	3 (13.6)	0	1		<0.001
	32–36	8 (4.1)	4 (18.2)	4 (2.3)	-	-	
	37–41	176 (90.7)	15 (68.2)	161 (93.6)	-	-	
	≥42	7 (3.6)	0	7 (4.1)	-	-	
Sex	Feminine	92 (47.4)	6 (27.3)	86 (50)	0.375	0.140–1.004	**0.044**
	Masculine	102 (52.6)	16 (72.7)	86 (50)	1		
Birth weight^o^	<1,500 g	5 (2.6)	4 (18.2)	1 (0.6)	1		
	1,500–2,499 g	18 (9.3)	7 (31.8)	11 (6.4)	0.172	0.003–2.220	0.263
	2,500 g–3,999 g	161 (83)	10 (45.5)	151 (87.8)	0.018	0.001–0.200	**<0.001**
	≥4,000 g	10 (5.2)	1 (4.5)	9 (5.2)	0.034	0.001–0.844	**0.034**
IUGR^p^	Yes	9 (4.6)	6 (27.3)	3 (1.7)	21.125	4.82–92.586	**<0.001**
	No	185 (95.4)	16 (72.7)	169 (98.3)	1		
Infectious risk^q^	Yes	50 (25.8)	14 (63.6)	36 (20.9)	6.611	2.574–16.978	**<0.001**
	No	144 (74.2)	8 (36.4)	136 (79.1)			
Congenital malformation	Yes	4 (2.1)	4 (18.2)	0	-	-	<0.001
	No	190 (97.9)	18 (81.8)	172 (100)	-	-	

ANC, antenatal care; CI, confidence interval; cOR: crude odds ratio; GA, gestational age; HAM: Hospital Dr. Ayres de Menezes; IPI, intestinal parasitic infection; IUGR, intrauterine growth restriction; PROM, prolonged rupture of membranes.

Bold values indicate significant *p*-value ≤0.05.

^a^ Urban residence for women living in the capital city (Água Grande) and rural areas in all other districts (Mé-Zochi, Cantagalo, Lobata, Lembá, Caué and Principe Island).

^b^ Poor birth spacing birth intervals of less than 2 years (42).

^c^ High-pregnancy risk is registered in the ANC pregnancy card if the current pregnancy is defined as one or more of the following: 1) pregnant women age less than 15 years old or greater than 35, 2) grand multipara for women with six or more labors, 3) previous history of a stillbirth or early neonatal death, 6) previous caesarean section, and 7) previous hemorrhagic complication.

^d^ Maternal disease was operationally defined as one or more of the following conditions during the current pregnancy: pre/eclampsia, gestational diabetes, malaria, bacteriuria, anemia, and intestinal parasitic infection.

^e^ Anaemia during pregnancy as a hemoglobin concentration <11 g/dl.

^f^ Glycaemia >105 mg/dl,.

^g^ Through a sickle cell solubility test, which involves treating a thin blood film with sodium dithionate under hypoxic conditions and observing for sickling under a light microscope, that is the screening technique available in STP and performed to pregnant women with anemia or clinical suspicion. A positive result can suggest either sickle cell anemia or the sickle cell trait (43).

^h^ Foetal malpresentation was determined if the presenting fetal part was noncephalic (e.g. breech, transverse, oblique) (32).

^i^ Prolonged rupture of membrane (PROM) was defined as a rupture of membrane lasting longer than 18 hours before labor began (34, 35).

^j^ Preeclampsia (hypertension ≥140/90 mmHg and proteinuria in dipsticks in women who were normotensive at ANC).

^k^ Obstructed labor was operationally defined as the sum of all cesarean sections due to mechanical problems or fetal distress and all instrumental delivery (37).

^l^ Postpartum hemorrhage was defined as >500 ml bleeding.

^m^ IVD in STP is only performed by vacuum.

^n^ Gestational age was estimated from the date of onset of the last normal menstrual period or through ultrasound dating of pregnancy. Prematurity was defined as a delivery before 37 complete weeks of gestation from the date of onset of the last normal menstrual period and subcategorized as very preterm (28 to 31 weeks) and moderate to late preterm (32 to 37 weeks).

^o^ Low birth weight was defined as a newborn weight less than 2,500 grams (up to and including 2,499 g) at birth regardless of gestational age (44). Low birth weight was further categorized into very low birth weight (VLBW, <1,500 g) (45). Low birth weight is a result of preterm birth, intrauterine growth restriction or both (45).

Large birth weight when ≥4,000 g (macrosomia) irrespective of gestational age.

^p^ There is still no consensual definition for intrauterine growth restriction (IUGR) or fetal growth restriction in Africa (46). In this study, it was defined as the term LBW (i.e., birthweight <2,500 g and gestational age ≥37 weeks of gestation) due to low *in utero* measurements through obstetric ultrasounds in the country (46).

^q^ Infectious risk was operationally defined as the sum of all the following risk factors: (1) maternal fever (axillary temperature >37.9 C) at the time of delivery, (2) prolonged rupture of membrane (≥18 h), and/or (3) foul-smelling amniotic fluid (47).

**Table 2:** Univariable logistic regression analysis of neonatal deaths among newborns who were live births *n* = 178 [death-outcome group: 6 and alive-outcome group: 172].

Variables	Categories	Total	Death-outcome group	Alive-outcome group	cOR	(95%) CI	*p* value
		*n* = 178^a^	*n* = 6^a^	*n* = 172			
			*n* (%)	*n* (%)			
Neonatal resuscitation	Yes	7 (3.9)	2 (33.3)	5 (2.9)	16.700	2.457–113.495	**0** **.** **004**
	No	171 (96.1)	4 (66.7)	167 (97.1)	1		** **
Fetal distress at birth^b^	Yes	26 (14.6)	5 (83.3)	21 (12.2)	35.952	4.004–322.859	**0** **.** **001**
	No	152 (85.4)	1 (16.7)	151 (87.8)	1		
Birth asphyxia^c^	Yes	10 (5.6)	4 (66.7)	6 (3.5)	55.333	8.421–363.600	**<0** **.** **001**
	No	168 (94.4)	2 (33.3)	166 (96.5)	1		** **
Admission at NCU	Yes	15 (8.4)	3 (50)	12 (7)	13.333	2.425–73.310	**0** **.** **003**
	No	163 (91.6)	3 (50)	160 (93)	1		

CI, confidence interval; cOR, crude odds ratio; NCU, neonatal care unit.

Bold values indicate significant *p*-value ≤0.05.

^a^ Stillbirths excluded.

^b^ Fetal distress was defined as a low Apgar score <7 at the first minute of life (score from 0 to <7) (26, 48).

^c^ Birth asphyxia was defined as a low Apgar score <7 at the fifth minute of life (score from 0 to <7) (26, 48).

There were no maternal deaths in this study, with a total of 2 maternal near-misses occurring in the stillbirths' motherś death-outcome group numbers 5 and 11 that needed hysterectomy intervention due to atonic uterus with major obstetric hemorrhage.

### Factors associated with perinatal and neonatal deaths

Crude odds ratios were estimated to assess the association of perinatal and neonatal deaths with several characteristics (Tables 1, 2). Results of this analysis showed that meconium-stained amniotic fluid, prolonged rupture of membranes, transfer from another unit, and instrumental vaginal delivery were significantly associated with perinatal and neonatal deaths.

### Sociodemographic factors

A maternal secondary education level (cOR 0.162, 95% CI 0.46–0.566, *p* = 0.002) and a babýs father secondary education level (cOR 0.140, 95% CI 0.029–0.67, *p* = 0.006) were found to be protective factors for perinatal and neonatal deaths.

### Antepartum factors

The odds of perinatal and neonatal deaths were three times higher among mothers classified as having a high-pregnancy risk (cOR 2.91, 95% CI 1.18–7.22, *p* = 0.017). Mothers without hemoglobin test during the ANC follow-up had a higher risk of having a perinatal and neonatal death (cOR 5.68, 95% CI 1.89–17.05, *p* = 0.004) than those without anemia.

### Health facility-related factors

The odds of perinatal and neonatal deaths were six times higher among mothers transferred from another unit compared to those directly admitted at HAM maternity (cOR 6.08, 95% CI 1.95–18.90, *p* = 0.004).

### Intrapartum factors

PROM as well as meconium-stained amniotic fluid were other intrapartum factors, with the odds of deaths being almost five times higher for newborns whose mothers had a PROM (cOR 4.84, 95% CI 1.47–15.93, *p* = 0.016), and four times higher for those with a meconium-stained amniotic fluid (cOR 4.38, 95% CI 1.74–10.98, *p* = 0.002).

For the mode of delivery, having an instrumental assisted delivery was associated with an eightfold higher risk of perinatal and neonatal death (cOR 8.90, 95% CI 1.68–47.21, *p* = 0.020).

### Newborns' factors

Newborns with intrauterine growth restriction had a twenty-one-fold higher risk of death (cOR 21.13, 95% CI 4.82–92.59, *p* < 0.001), and newborns with an infectious risk had almost sevenfold higher odds of dying (cOR 6.61, 95% CI 2.57–16.98, *p* < 0.001). Regarding newborn characteristics, female neonates (cOR 0.38, 95% CI 0.14–1.00, *p* = 0.044) and birth weight greater than 2,500 g and lower than 3,999 g (cOR 0.017, 95% CI 0.002–0.162, *p* < 0.001), and greater than 4,000 g (cOR 0.028, 95% CI 0.001–0.564, *p* = 0.034) were protective factors against perinatal and neonataldeaths.

Postpartum characteristics were only assessed for a total of six death-outcome group since sixteen stillbirths were not further included for analysis. Performance of neonatal resuscitation, fetal distress at birth (APGAR score at first-minute inferior to seven), birth asphyxia, and admission to the neonatal care unit were all related to an increased risk for neonatal death (Table 2).

## Discussion

In this prospective cohort study, the main aim was to identify associated factors with perinatal and neonatal death. The most vulnerable period for death—partum, early or late neonatal period—and to identify where they die—in the maternity, at home or in the pediatric ward—was also possible as we were able to follow newborns up until their 28th day of life. In this study, we found that 90% died in HAM maternity before the 7th day of life, that is, during the perinatal period, with mainly being stillborn (73%). The magnitude of stillbirths observed in this study (3%) is in line with the studies conducted in Nigeria (4.8%) (49) and Tanzania (3.5%) (50) and lower compared with studies from Ethiopia (6.7%) (51). Most stillbirths were intrapartum deaths, as also identified in LMICs similar to STP, and most could have been prevented (52, 53).

Thus, this study indicates a probability of a stillbirth in 30 per 1,000 liverbirths, and a probability of neonatal death rate of 11 per 1,000 livebirths. The value found for the perinatal mortality is two times higher than the rates estimated for the country (18, 19).

In this study, intrapartum characteristics, such as pregnant women with meconium-stained amniotic fluid, prolonged rupture of membranes, and instrumental vaginal delivery, were the main factors significantly associated with perinatal and neonatal death. Health facility-related factors, as pregnant women transferred from another unit were also at a significant risk.

High-pregnancy risk score notification was the only antepartum factor significantly associated with perinatal and neonatal deaths. For newborns, experiencing fetal distress at birth, needing resuscitation maneuvers, having birth asphyxia and admission to the neonatal unit were identified as high-risk factors for death. Being a female newborn and having a birth weight greater than 2,500 grams were found to be protective factors.

Odds of experiencing death were identified as four times higher among newborns from mothers with meconium-stained amniotic fluid. This finding is consistent with studies from Ethiopia (6, 54) and Yemen (13) that also reported higher rates of fetal death due to intrauterine passage of meconium into amniotic fluid and a fivefold increase in perinatal mortality compared with low-risk patients with clear amniotic fluid (55). Meconium-stained amniotic fluid is associated with fetal distress since the fetus, in response, inhales the meconium, which in turn leads to airway obstruction, surfactant dysfunction and pneumonitis, which leads to loss of the fetus (6).

PROM was also a significant factor associated with neonatal deaths in this study, showing fivefold higher odds for the death-outcome group. This association was also identified in other studies from low-resource constrained countries reporting one-third of stillbirths occurring during labor as a result of prolonged labor or obstructed labor not attended to promptly (38, 39, 41, 56, 57). The reason for this might be because PROM is a risk factor for early-onset neonatal sepsis as well as a high risk of fetal distress, respiratory distress syndrome, intraventricular hemorrhage, and death (34, 35, 58). In this study we found an overall prevalence of PROM of 8.1%, which is in accordance with the global incidence that ranges from approximately 5% to 10% of all deliveries in the world (59, 60).

Lack of emergency obstetric care (EmOC) is well-known to increase the risk of neonatal mortality, as laboring mothers with complications cannot immediately receive appropriate health services, such as access to a cesarean section (39). In this study, mothers who needed to be transferred from another health unit for delivery at HAM had a sixfold higher risk of having a neonatal death outcome than mothers directly admitted at HAM. Half the mothers who were transferred from the death-outcome group had to do a 60 -kilometer journey that took approximately two to three hours until reaching HAM maternity in the capital city. Distance from a health facility with EmOC and the considerable travel times are well-known barriers and influential factors of birth adverse outcomes also found in other studies in sub-Saharan Africa (58, 61).

In this study, the overall rate of 3.1% of instrumental assisted vaginal birth deliveries was found to be lower compared to 17% of births by cesarean section and 79.9% of normal vaginal deliveries. Instrumental-assisted deliveries are an effective intervention for deliveries complicated by prolonged labor or fetal distress but are also related to adverse outcomes since fetal distress or cephalopelvic disproportion are more frequently the motive to use this technique. Therefore, the ninefold higher risk of death outcome found in this study, similar to other researchers reporting (14, 62, 63), relates to late appropriate intrapartum care intervention with subsequently higher rates of fetal distress and risk of an intrapartum stillbirth.

Antenatal care health services in STP follow a high-risk stratification for each pregnancy and, in this study, this notification of a high-risk pregnancy in the pregnant women antenatal pregnancy card was significantly associated with a threefold higher risk for fetal and neonatal death than alive-outcome group. High-pregnancy risk was operationally defined as one or more of the following according to STP national practice, namely, age as inferior to 15 years old or superior to 35, grand multipara, previous history of a stillbirth or early neonatal death, previous cesarean section, and previous hemorrhagic complication. This is consistent with other studies that reported higher rates of perinatal and neonatal mortality with extreme maternal ages and previous adverse obstetric history (grand multipara, previous stillbirth, or early neonatal death) (1, 13). However, in this study, we could not find a statistically significant difference between groups when each of these variables were independently analyzed.

Regarding the newborns' characteristics, the overall sex ratio at birth was 52.6% for males and 47.4% for females. The death rate was higher in male neonates (72%) than in the female death-outcome group (27.3%), with female sex being a protective factor. Sex variations are frequently reported, with a highly consistent pattern of excess male mortality across different populations and income groups (64). Male infants are more vulnerable to fetal and early neonatal death, with most studies reporting no gender difference in mortality after 7 days of age (64). This sex difference may be explained by a sex-specific difference in the growth and function of male and female placentae, making boys more vulnerable to different adverse outcomes (64–66).

Birth weights of more than 2,500 g were protective factors for death outcome in this study. This finding is comparable with different research on other African countries that associates a birth weight of less than 2,500 g (LBW) with higher rates of stillbirth and neonatal death (1). Additionally, the odds of experiencing death were 21 times higher for babies with IUGR or fetal growth restriction defined, in this study, as a term LBW (birthweight <2,500 g and gestational age ≥37 weeks) (46). Low birth weight is a worldwide recognized important multifaceted public health problem since LBW infants are 20 times more likely to develop complications and die than normal weight infants (6, 67).

We found that the odds of death were six times higher for newborns with maternal infectious risk. This finding is in agreement with previous studies conducted in SSA, as neonatal infectious risk is linked to amniotic fluid contamination that can cause intrapartum fetal infection or early postpartum neonatal infection with sepsis complicated with septic shock and multiple organ dysfunction, in which both are the most common causes of death in the perinatal period (38, 47). Infection is an important cause of stillbirth and neonatal death in LMICs, although there is a lack of overall information regarding the organisms involved, the types of transmission, and the mechanisms of death in these constrained countries (68). In Sao Tome & Principe, malaria and syphilis are no longer an infection-burden and cause of stillbirth or neonatal death; therefore, other bacterial and viral maternal infections should be linked to stillbirth and ENND (68). In STP, there are no means to establish a neonatal infection through blood cultures; that is, neonatal infection is based only on clinical signs with no bacteriological documentation. Additionally, nothing is known about the rate of vertical transmission of Streptococcus Group B (GBS) from colonized mothers at birth in the country. Therefore, there is a current gap in the knowledge, capability of making diagnosis and prevention and treatment of neonatal infections in STP. For instance, a study from Ethiopia (68) detected that the rate of vertical transmission of GBS from colonized mothers at birth was as high as 45.02% due to term PROM, PROM ≥18 h before delivery and mothers having fever during labor. Detecting the risk for vertical transmission of GBS and implementing prevention methods such as adequate intrapartum administration of antibiotics are recognized as easy and affordable practices for reducing mortality due to infectious causes and should be implemented in the country along with culture techniques (47).

All four congenital major malformations detected in this study resulted in a perinataldeath, although due to the low number of newborns enrolled it was not possible to establish a statistically significant difference. Congenital malformations are known to be associated with poor outcomes for newborns, and 10%–20% of stillbirths are attributed to intrinsic fetal anomalies (57). Studies from Zimbabwe (69), Ethiopia (1) and Yemen (13) found that congenital anomalies among stillbirths were associated with a 5-fold (69), 34-fold (1) and 40-fold (13) higher risk than those among live births. Another important consideration regarding congenital anomalies is the type of malformation, since some, such as neural tube defects, are preventable and can be reduced by 30 to 50% by folic acid supplementation in pregnant women and have been estimated to cause 29% of deaths related to congenital anomalies in LMICs (4). In this study, three out of the four major anomalies were neural tube defects, highlighting the need to enhance proper supplementation to preconceptional women in the country.

In this study, we were not able to find a higher risk associated with newborn gestational age. The high rate of stillbirths found in our study as well as the low number of preterm babies enrolled (5.6%) explain the lack of association. Stillbirths are among the most common pregnancy-related adverse outcomes worldwide, but they differ between low- and high-income countries. In high-income countries, most stillbirths occur early in the preterm period, whereas in low-resource constrained countries, most occur in term or in late preterm births, as found in our study (70). Additionally, the neonatal deathis higher in babies born between 37 and 38 weeks of gestation than in those born between 39 and weeks (4).

From stillbirths in this study, 69% occurred during the intrapartum period, in accordance with other studies from LMICs (5). These intrapartum stillbirths mean that intrauterine death occurred after the onset of labor and before birth (fresh stillbirth). We can also guess that some of them could be early neonatal deaths, as there are known barriers and difficulties in these contexts to establish whether a fetus or motionless newborn is living or dead after its delivery. For instance, information on some Apgar scores in these death-outcome group was not recorded, perhaps because of lack of time, especially when the neonates had to be rushed to receive resuscitation maneuvers. Studies from SSA highlight that it is very frequent that some depressed but living fetuses with a possible heartbeat do not receive resuscitation maneuvers and are prompt classified as stillbirths (1, 70). This is supported by a systematic review of sixteen hospitals and community-based perinatal mortality studies (71, 72).

In this study, newborns who needed resuscitation maneuvers were at a 16-fold higher risk of dying. Additionally, the odds of neonatal death from fetal distress at birth and birth asphyxia were 36 and 55 times higher, respectively. These results are in line with studies conducted in Cameroon (34) and Ethiopia (39) as well as published literature that suggests that lower APGAR scores are associated with severe multiorgan damage resulting in brain damage, lung dysfunction, cardiomyopathy, renal failure, hepatic failure, necrotizing enterocolitis and consequently death (73). Previous studies conducted by the authors, that compared outcomes between adolescent pregnant girls and older counterparts identified that adverse outcomes imputable to adolescent births were fetal distress and performance of neonatal resuscitation maneuvers, highlighting the risks surrounding this period among deliveries at HAM maternity unit (25, 26).

Perinatal asphyxia can be caused by factors grouped according to whether they are before birth (antepartum risk actors), during birth (intrapartum risk factors), or after birth (postpartum or fetal risk factors) (48, 73). Nonetheless, the single most important predictor is, undoubtedly, the quality of intrapartum care during labor and delivery.

The most important aspect of this study for public health is that it identifies potential characteristics that predispose newborns to life-threatening conditions, which is critical to address the underlying causes and provide prompt interventions by various stakeholders in the healthcare system (74).

This study allows us to perceive how to answer our question “when, where and why do newborns die in STP?” as “when”: mostly during labor, “where”: in uterus and “why”: mainly due to fetal distress and intrapartum-related deaths probably due to low quality and constraints of care during labor and delivery. Ending preventable stillbirths and neonatal deaths does not necessarily require new or innovative interventions. Most modifiable risk factors identified in this study can be addressed and prevented with timely, quality care during childbirth, including ongoing intrapartum monitoring and opportune intervention in case of complications (5). Some interventions, such as cardiotocography to monitor a baby's well-being in the womb by measuring contractions, are estimated to reduce the rate of infant deaths around the time of birth by 80% (75). Thus, according to this study results, the priority for STP is to prevent stillbirths through proper fetal surveillance, therefore, measures such as, the implementation of simple techniques in all maternity units, namely foetal heart rate monitoring and to provide algorithms to deliver prompt interventions when needed are essential.

In summary, the findings of this study will be useful to health policymakers and program developers in implementing appropriate interventions to achieve the newborn health post-2015 Sustainable Development Goals of no more than 12 neonatal deaths per 1,000 live births in Sao Tome & Principe by 2030 (76).

### Strengths and limitations

In this study, the researcher retrieved maternal and neonatal data directly from ANC cards and maternity registers to limit recall bias. The selection of the death-outcome group and alive-outcome group was based on the records of maternal and neonatal registers; therefore, it is less likely that this study has misclassification biases both in the exposure and death-outcome group ‒alive-outcome group categories (44, 67).

Regarding the limitations, this is a relatively small study aiming to identify factors associated with perinatal and neonatal deaths in Sao Tome & Principe with a cohort of 194 newborns that were followed up until 28th days of age, 22 died and 172 survived. Thus, this study results cannot be generalized.

Another limitation is that some of the variables mentioned in the univariable analysis had wide confidence intervals and high odds ratios due to the low number of newborns in the death-outcome group enrolled in this study.

Nonetheless, the current study can assist Sao Tome & Principe policy makers and stakeholders in designing new policies for the country to improve maternal and neonatal health outcomes.

## Conclusions

Perinatal mortality is a major public health problem in Sao Tome & Principe, as reinforced by this study, indicating a probability of a stillbirth in 30 per 1,000 liverbirths, and a probability of neonatal death rate of 11 per 1,000 livebirths, values higher than the rates estimated for the country.

Complications such as a high-risk pregnancy score, meconium-stained amniotic fluid, prolonged rupture of membranes, being transferred from another unit, and an instrumental-assisted vaginal delivery increased the risk of stillbirth and neonatal death between 4– and 9–fold, and 90% of all these deaths occurred in the perinatal period.

Newborns with an infectious risk, intrauterine growth restriction, fetal distress at birth, who needed resuscitation maneuvers, birth asphyxia, and those admitted to the neonatal unit had a 3- to 55-fold higher risk for dying than the alive-outcome group. Female newborn and birth weight of more than 2,500 g were found to be protective factors.

Thus, the priority for STP is to prevent stillbirths through proper fetal surveillance. Measures such as, the implementation of simple techniques in all maternity units, namely fetal heart rate monitoring and to provide algorithms to deliver prompt interventions when needed will improve perinatal and neonatal outcomes and survival in Sao Tome & Principe.

## Data Availability

The original contributions presented in the study are included in the article/Supplementary Material, further inquiries can be directed to the corresponding author.

## References

[1] Getiye Y Fantahun M. Factors associated with perinatal mortality among public health deliveries in Addis Ababa, Ethiopia, an unmatched case control study. BMC Pregnancy Childbirth. (2017) 17(1):1–7. 10.1186/s12884-017-1420-7 28747161 PMC5530490

[2] Pathirana J Muñoz FM Abbing-Karahagopian V Bhat N Harris T Kapoor A Neonatal death: case definition & guidelines for data collection, analysis, and presentation of immunization safety data. Vaccine. (2016) 34(49):6027–37. 10.1016/j.vaccine.2016.03.040 27449077 PMC5139812

[3] WHO. Every New Born: An Action Plan to End Preventable Deaths. Geneva: WHO Press, World Health Organization (2014). Available online at: https://cdn.who.int/media/docs/default-source/mca-documents/advisory-groups/quality-of-care/every-new-born-action-plan-(enap).pdf?sfvrsn=4d7b389_2

[4] Lehtonen L Gimeno A Parra-Llorca A Vento M. Early neonatal death: a challenge worldwide. Semin Fetal Neonatal Med. (2017) 22(3):153–60. 10.1016/j.siny.2017.02.006 28238633

[5] Hug L You D Blencowe H Mishra A Wang Z Fix MJ Global, regional, and national estimates and trends in stillbirths from 2000 to 2019: a systematic assessment. Lancet. (2021) 398(10302):772–85. 10.1016/S0140-6736(21)01112-0 34454675 PMC8417352

[6] Dagne HM Melku AT Abdi AA. Determinants of stillbirth among deliveries attended in bale zone hospitals, Oromia regional state, Southeast Ethiopia: a case–control study. Int J Women’s Health. (2021) 13:51. 10.2147/IJWH.S276638 33447092 PMC7802824

[7] Lawn JE Kerber K Enweronu-Laryea C Massee Bateman O. Newborn survival in low resource settings—are we delivering? BJOG: An Int J Obstet Gynaecol. (2009) 116:49–59. 10.1111/j.1471-0528.2009.02328.x 19740173

[8] Lawn JE Cousens S Zupan J. Lancet neonatal survival steering team. 4 million neonatal deaths: when? where? why? Lancet. (2005) 365(9462):891–900. 10.1016/S0140-6736(05)71048-5 15752534

[9] Lawn JE Blencowe H Pattinson R Cousens S Kumar R Ibiebele I Stillbirths: where? when? why? how to make the data count? Lancet. (2011) 377(9775):1448–63. 10.1016/S0140-6736(10)62187-3 21496911

[10] Blencowe H Cousens S. Addressing the challenge of neonatal mortality. Trop Med Int Health. (2013) 18(3):303–12. 10.1111/tmi.12048 23289419

[11] Lavin T Allanson ER Nedkoff L Preen DB Pattinson RC. Applying the international classification of diseases to perinatal mortality data, South Africa. Bull W H O. (2018) 96(12):806. 10.2471/BLT.17.206631 30505028 PMC6249699

[12] Tavares Da Silva F Gonik B McMillan M Keech C Dellicour S Bhange S Stillbirth: case definition and guidelines for data collection, analysis, and presentation of maternal immunization safety data. Vaccine. (2016) 34(49):6057–68. 10.1016/j.vaccine.2016.03.044 27431422 PMC5139804

[13] Obad MA Taher R Qayad M Khader YS. Risk factors of stillbirth in Yemen. J Neonatal Perinatal Med. (2018) 11(2):131–6. 10.3233/NPM-18174618 29843265

[14] Adane AA Ayele TA Ararsa LG Bitew BD Zeleke BM. Adverse birth outcomes among deliveries in Gonder university hospital, Northwest Ethiopia. BMC Pregnancy Childbirth. (2014) 14:90. 10.1186/1471-2393-14-90 24576205 PMC3996071

[15] Chi BH Wang L Read JS Taha TE Sinkala M Brown ER Predictors of stillbirth in sub-saharan Africa. Obstet Gynecol. (2007) 110(5):989–97. 10.1097/01.AOG.0000281667.35113.a5 17978109

[16] Stringer EM Vwalika B Killam WP Giganti MJ Mbewe R Chi BH Determinants of stillbirth in Zambia. Obstet Gynecol. (2011) 117(5):1151–9. 10.1097/AOG.0b013e3182167627 21508755

[17] Sao Tome and Principe WHO statistical profile. WHO Libr. (2015). Available online at: https://www.who.int/gho/countries/stp.pdf (accessed May 8, 2023).

[18] INE e UNICEF. Inquérito de Indicadores Múltiplos 2019, Relatório final. São Tomé, São Tomé e Príncipe: Instituto Nacional de Estatística e Fundo das Nações Unidas para a Infância (2020). Available online at: https://mics-surveys-prod.s3.amazonaws.com/MICS6/West%20and%20Central%20Africa/Sao%20Tome%20and%20Principe/2019/Survey%20findings/Sao%20Tome%20e%20Principe%202019%20MICS%20Survey%20Findings%20Report_Portuguese.pdf (accessed May 8, 2023).

[19] UNICEF IN de E (INE) e. Inquérito de Indicadores Múltiplos, São Tome e Principe, MICS-STP, 2014, Principais resultados. SaoTome, SaoTome e Principe, INE e UNICEF. 2015; Instituto Nacional de Estatística, 2016. Inquérito aos Indicadores Múltiplos 2014 de São Tomé e Príncipe, Relatório Final. São Tomé, São Tomé e Príncipe. Available online at: http://ms.gov.st/wp-content/uploads/2019/01/MICS-Final-Report-STP_Portugu%C3%AAs.Pdf (accessed May 8, 2023).

[20] Ministério da Saúde_ República Democrática de Sao Tome e Principe_Plano Nacional do Desenvolvimento da Saúde 2017–2021.

[21] Bhutta Z Das JK Bahl R Lawn JE Salam R Paul VK Can available interventions end preventable deaths in mothers, newborn babies, and stillbirths, and at what cost? Lancet. (2014) 384(9940):347–70. 10.1016/S0140-6736(14)60792-3 24853604

[22] Beyond 2015, Gcap, Ifp. Civil Society Demands for the Post-2015 Agenda from 39 Countries (2013).

[23] Dickson KE Simen-Kapeu A Kinney MV Huicho L Vesel L Lackritz E Every newborn: health-systems bottlenecks and strategies to accelerate scale-up in countries. Lancet. (2014) 384(9941):438–54. 10.1016/S0140-6736(14)60582-1 24853600

[24] Nair N Tripathy P Prost A Costello A Osrin D. Improving newborn survival in low-income countries: community-based approaches and lessons from South Asia. PLoS Med. (2010) 7(4):e1000246. 10.1371/journal.pmed.1000246 20386728 PMC2850383

[25] Vasconcelos A Bandeira N Sousa S Pereira F Machado MD. Adolescent pregnancy in Sao Tome and Principe: a cross-sectional hospital-based study. BMC Pregnancy Childbirth. (2022) 22(1):1–5. 10.1186/s12884-022-04632-z 35428214 PMC9013095

[26] Vasconcelos A Bandeira N Sousa S Machado MC Pereira F. Adolescent pregnancy in Sao Tome and Principe: are there different obstetric and perinatal outcomes? BMC Pregnancy Childbirth. (2022) 22(1):453. 10.1186/s12884-022-04779-9 35642050 PMC9153156

[27] Vasconcelos A Sousa S Bandeira N Baptista JL Machado MD Pereira F. PO 8592 Why, when and where do newborns not only get sick but also die in São tomé and Príncipe? A case-control study. BMJ Global Health. (2019) 4(Suppl 3):A60.

[28] Vasconcelos A Sousa S Bandeira N Alves M Papoila AL Pereira F Antenatal screenings and maternal diagnosis among pregnant women in Sao Tome & Principe-Missed opportunities to improve neonatal health: a hospital-based study. PLOS Global Public Health. (2022) 2. 10.1371/journal.pgph.0001444 36962895 PMC10021443

[29] Vasconcelos A Sousa S Bandeira N Alves M Papoila AL Pereira F Intestinal parasitic infections, treatment and associated factors among pregnant women in Sao Tome and Principe: a cross-sectional study. J Trop Med. (2022) 2022:7492020. 10.1155/2022/7492020 36438179 PMC9699776

[30] Vasconcelos A Sousa S Bandeira N Alves M Papoila AL Pereira F Determinants of antenatal care utilization—contacts and screenings—in Sao Tome & Principe: a hospital-based cross-sectional study. Arch Public Health. (2023) 81:107. 10.1186/s13690-023-01123-1 37328871 PMC10273617

[31] Vasconcelos A Sousa S Bandeira N Alves M Papoila AL Pereira F Adverse birth outcomes and associated factors among newborns delivered in Sao Tome & Principe: a case–control study. PloS One. (2023). 10.1371/journal.pone.0276348 PMC10328319 37418369

[32] Mulowooza J Santos N Isabirye N Inhensiko I Sloan NL Shah S Midwife-performed checklist and ultrasound to identify obstetric conditions at labour triage in Uganda: a quasi-experimental study. Midwifery. (2021) 96:102949. 10.1016/j.midw.2021.102949 33631411 PMC7988503

[33] Weissmann-Brenner A Meyer R Domniz N Levin G Hendin N Yoeli-Ullman R The perils of true knot of the umbilical cord: antepartum, intrapartum and postpartum complications and clinical implications. Arch Gynecol Obstet. (2022) 305(3):573–9. 10.1007/s00404-021-06168-7 34405285

[34] Chiabi A Takou V Mah E Nguefack S Siyou H Takou V Risk factors for neonatal mortality at the yaounde gynaeco-obstetric and pediatric hospital. Cameroon. Iran J Pediatr. (2014) 24(4):393–400. 25755860 PMC4339562

[35] Tewabe T Mohammed S Tilahun Y Melaku B Fenta M Dagnaw T Clinical outcome and risk factors of neonatal sepsis among neonates in felege hiwot referral hospital, bahir dar, amhara regional state, North West Ethiopia 2016: a retrospective chart review. BMC Res Notes. (2017) 10:265. 10.1186/s13104-017-2573-1 28693597 PMC5504561

[36] Avagliano L Massa V Bulfamante G. Meconium-stained amniotic fluid and histologic signs of fetal distress in stillbirths. Eur J Obstet Gynecol Reprod Biol. (2021) 266:55–62. 10.1016/j.ejogrb.2021.09.016 34592650

[37] Shaikh S Shaikh AH Shaikh SA Isran B. Frequency of obstructed labor in teenage pregnancy. Nepal J Obstet Gynaecol. (2012) 7(1):37–40. 10.3126/njog.v7i1.8834

[38] Alemu AY Belay GM Berhanu M Minuye B. Determinants of neonatal mortality at neonatal intensive care unit in Northeast Ethiopia: unmatched case-control study. Trop Med Health. (2020) 48(1):1–0. 10.1186/s41182-019-0188-z 32514229 PMC7268585

[39] Yego F D’Este C Byles J Nyongesa P Williams JS. A case-control study of risk factors for fetal and early neonatal deaths in a tertiary hospital in Kenya. BMC Pregnancy Childbirth. (2014) 14(1):1–9. 10.1186/1471-2393-14-1 25432735 PMC4298961

[40] Demisse AG Alemu F Gizaw MA Tigabu Z. Patterns of admission and factors associated with neonatal mortality among neonates admitted to the neonatal intensive care unit of university of Gondar hospital, Northwest Ethiopia. Pediatric Health Med Ther. (2017) 8:57. 10.2147/PHMT.S130309 29388628 PMC5774602

[41] Abadiga M Mosisa G Tsegaye R Oluma A Abdisa E Bekele T. Determinants of adverse birth outcomes among women delivered in public hospitals of Ethiopia, 2020. Arch Public Health. (2022) 80(1):1–7. 10.1186/s13690-021-00776-0 34983656 PMC8728986

[42] Pimentel J Ansari U Omer K Gidado Y Baba MC Andersson N Factors associated with short birth interval in low- and middle-income countries: a systematic review. BMC Pregnancy Childbirth. (2020) 20:156. 10.1186/s12884-020-2852-z 32164598 PMC7069040

[43] Esoh K Wonkam-Tingang E Wonkam A. Sickle cell disease in sub-saharan Africa: transferable strategies for prevention and care. Lancet Haematol. (2021) 8(10):e744–55. 10.1016/S2352-3026(21)00191-5 34481550

[44] Kikuchi K Ansah EK Okawa S Enuameh Y Yasuoka J Nanishi K Effective linkages of continuum of care for improving neonatal, perinatal, and maternal mortality: a systematic review and metaanalysis. PloS One. (2015) 10(9):e0139288. 10.1371/journal.pone.0139288 26422685 PMC4589290

[45] Cutland CL Lackritz EM Mallett-Moore T Bardají A Chandrasekaran R Lahariya C Low birth weight: case definition & guidelines for data collection, analysis, and presentation of maternal immunization safety data. Vaccine. (2017) 35(48Part A):6492. 10.1016/j.vaccine.2017.01.049 29150054 PMC5710991

[46] Accrombessi M Zeitlin J Massougbodji A Cot M Briand V. What do we know about risk factors for fetal growth restriction in Africa at the time of sustainable development goals? A scoping review. Paediatr Perinat Epidemiol. (2018) 32(2):184–96. 10.1111/ppe.12433 29253317

[47] Huynh BT Kermorvant-Duchemin E Herindrainy P Padget M Rakotoarimanana FM Feno H Bacterial infections in neonates, Madagascar, 2012–2014. Emerg Infect Dis. (2018) 24(4):710–7. 10.3201/eid2404.161977 29553312 PMC5875286

[48] Wosenu L Worku AG Teshome DF Gelagay AA. Determinants of birth asphyxia among live birth newborns in university of Gondar referral hospital, northwest Ethiopia: a case-control study. PLoS One. (2018) 13(9):e0203763. 10.1371/journal.pone.0203763 30192884 PMC6128623

[49] Mbachu II Achigbu KI Odinaka KK Eleje GU Osuagwu IK Osim VO. Tracking stillbirths by referral pattern and causes in a rural tertiary hospital in Southern Nigeria. Niger Postgrad Med J. (2018) 25(2):87–93. 10.4103/npmj.npmj_73_18 30027919

[50] Chuwa FS Mwanamsangu AH Brown BG Msuya SE Senkoro EE Mnali OP Maternal and fetal risk factors for stillbirth in Northern Tanzania: a registry-based retrospective cohort study. PLoS One. (2017) 12(8):e0182250. 10.1371/journal.pone.0182250 28813528 PMC5557599

[51] Mulatu T Debella A Feto T Dessie Y. Determinants of stillbirth among women who gave birth at Hiwot Fana specialized university hospital, Eastern Ethiopia: a facility-based cross-sectional study. SAGE Open Med. (2022) 10:20503121221076370. 10.1177/2050312122107637 35154742 PMC8832588

[52] Chaibva BV Olorunju S Nyadundu S Beke A. Adverse pregnancy outcomes, “stillbirths and early neonatal deaths” in Mutare district, Zimbabwe. BMC Pregnancy Childbirth. (2019) 19:86. 10.1186/s12884-019-2229-3 30841873 PMC6402130

[53] Lawn JE Blencowe H Waiswa P Amouzou A Mathers C Hogan D Stillbirths: rates, risk factors, and acceleration towards 2030. Lancet. (2016) 387(10018):P587–603. 10.1016/S0140-6736(15)00837-5 26794078

[54] Mamo SA Teshome GS Tesfaye T Goshu AT. Perinatal asphyxia and associated factors among neonates admitted to a specialized public hospital in South Central Ethiopia: a retrospective cross-sectional study. PloS One. (2022) 17(1):e0262619. 10.1371/journal.pone.0262619 35025979 PMC8758104

[55] Desai D Maitra N Patel P. Fetal heart rate patterns in patients with thick meconium staining of amniotic fluid and its association with perinatal outcome. Int J Reprod Contracept Obstet Gynecol. (2017) 6(3):1030–5. 10.18203/2320-1770.ijrcog20170579

[56] Asiki G Baisley K Newton R Marions L Seeley J Kamali A Adverse pregnancy outcomes in rural Uganda (1996–2013): trends and associated factors from serial cross-sectional surveys. BMC Pregnancy Childbirth. (2015) 15:279. 10.1186/s12884-015-0708-8 26515763 PMC4627380

[57] Lawn J Shibuya K Stein C. No cry at birth: global estimates of intrapartum stillbirths and intrapartum-related neonatal deaths. Bull World Health Organ. (2005) 83:409–17. 15976891 PMC2626256

[58] Geleto A Chojenta C Musa A Loxton D. Barriers to access and utilization of emergency obstetric care at health facilities in sub-Saharan Africa: a systematic review of literature. Syst Rev. (2018) 7:183. 10.1186/s13643-018-0842-2 30424808 PMC6234634

[59] Assefa NE Berhe H Girma F Berhe K Berhe YZ Gebreheat G Risk factors of premature rupture of membranes in public hospitals at Mekele city, Tigray, a case control study. BMC Pregnancy Childbirth. (2018) 18:386. 10.1186/s12884-018-2016-6 30268103 PMC6162906

[60] Alam MM Saleem AF Shaikh AS Munir O Qadir M. Neonatal sepsis following prolonged rupture of membranes in a tertiary care hospital in Karachi, Pakistan. J Infect Dev Ctries. (2014) 8(01):067–73. 10.3855/jidc.3136 24423714

[61] Habte A Wondimu M. Determinants of maternal near miss among women admitted to maternity wards of tertiary hospitals in Southern Ethiopia, 2020: a hospital-based case-control study. PloS One. (2021) 16(5):e0251826. 10.1371/journal.pone.0251826 33999941 PMC8128231

[62] Thakur N Sunny AK Gurung R Basnet O Litorp H Ashish KC. Rate and neonatal outcomes among instrument assisted vaginal birth in 12 public hospitals in Nepal. Preprint. (2020). 10.21203/rs.3.rs-36775/v1

[63] Hailemichael HT Debelew GT Alema HB Weldu MG Misgina KH. Determinants of adverse birth outcome in Tigrai region, North Ethiopia: hospital-based case-control study. BMC Pediatr. (2020) 20(1):1–9. 10.1186/s12887-019-1835-6 31914947 PMC6947822

[64] Mondal D Galloway TS Bailey TC Mathews F. Elevated risk of stillbirth in males: systematic review and meta-analysis of more than 30 million births. BMC Med. (2014) 12(1):1. 10.1186/s12916-014-0220-4 PMC4245790 25428603

[65] Aghai ZH Goudar SS Patel A Saleem S Dhaded SM Kavi A Gender variations in neonatal and early infant mortality in India and Pakistan: a secondary analysis from the global network maternal newborn health registry. Reprod Health. (2020) 17(3):1. 10.1186/s12978-020-01028-0x 33334358 PMC7745348

[66] Pongou R. Why is infant mortality higher in boys than in girls? A new hypothesis based on preconception environment and evidence from a large sample of twins. Demography. (2013) 50(2):421–44. 10.1007/s13524-012-0161-5 23151996

[67] Anil KC Basel PL Singh S. Low birth weight and its associated risk factors: health facility-based case-control study. PloS One. (2020) 15(6):e0234907. 10.1371/journal.pone.0234907 32569281 PMC7307746

[68] Yadeta TA Worku A Egata G Seyoum B Marami D Berhane Y. Vertical transmission of group B streptococcus and associated factors among pregnant women: a cross-sectional study, eastern Ethiopia. Infect Drug Resist. (2018) 11:397. 10.2147/IDR.S150029 29559801 PMC5856028

[69] El T Gombe N Shambira G Chadambuka A Mufuta T Zizhou S. Determinants of perinatal mortality in Marondera district, Mashonal and East Province of Zimbabwe, 2009. Pan AFr Med J. (2011) 8(1). 10.4314/pamj.v8i1.71054 PMC3201615 22121416

[70] Goldenberg RL Harrison MS McClure EM. Stillbirths: the hidden birth asphyxia—US and global perspectives. Clin Perinatol. (2016) 43(3):439–53. 10.1016/j.clp.2016.04.004 27524446

[71] Gutman A Harty T O’Donoghue K Greene R Leitao S. Perinatal mortality audits and reporting of perinatal deaths: systematic review of outcomes and barriers. J Perinat Med. (2022) 50(6):684–712. 10.1515/jpm-2021-0363 35086187

[72] Berhan Y Berhan A. Perinatal mortality trends in Ethiopia. Ethiop J Health Sci. (2014) 24:29–40. 10.4314/ejhs.v24i0.4S 25489181 PMC4249204

[73] American College of Obstetricians and Gynecologists. The apgar score (committee opinion No. 644). Obstet Gynecol. (2015) 126(4):52–5. 10.1097/AOG.0000000000001108 26393460

[74] Habte A Lukas K Melis T Tamene A Sahle T Hailu M Determinants of neonatal near miss among neonates admitted to public hospitals in Southern Ethiopia, 2021: a case-control study. PLoS One. (2022) 17(5):e0268041. 10.1371/journal.pone.0268041 35522663 PMC9075625

[75] Ota E da Silva Lopes K Middleton P Flenady V Wariki WM Rahman MO Antenatal interventions for preventing stillbirth, fetal loss and perinatal death: an overview of cochrane systematic reviews. Cochrane Database Syst Rev. (2020) 12:CD009599. 10.1002/14651858.CD009599.pub2 33336827 PMC8078228

[76] Paulson KR Kamath AM Alam T Bienhoff K Abady GG Abbas J Global, regional, and national progress towards sustainable development goal 3.2 for neonatal and child health: all-cause and cause-specific mortality findings from the global burden of disease study 2019. Lancet. (2021) 398(10303):870–905. 10.1016/S0140-6736(21)01207-1 34416195 PMC8429803

