# Supplementary material for: Factors associated with perinatal and neonatal deaths in Sao Tome & Principe: a prospective cohort study
Source: Front Pediatr. 2024 Feb 16;12:1335926. doi: 10.3389/fped.2024.1335926 (PMC10904650; doi:10.3389/fped.2024.1335926)
Supplement: Supplementary file 1 [file Datasheet1.pdf]

Additional file 1 Death-outcome group**:** characteristics of the stillbirths (n=16)

Stillbirth number	Intrapartum stillbirths	Gestational age range[ǂ]	sex	Birth weight	Major malformation	Pregnant Transferred	Reason for transferred	PROM	Meconium	Eclampsia	Atonic uterus	Birth attendant	Dystocia
1	no	4	male	3100	yes*	yes[1]	no fetus heartbeat	-	yes	-	-	midwife	no
2	no	1	male	1200	-	yes[1]	no fetus heartbeat	-	-	-	-	midwife	no
3	no	4	male	2000	-	yes[2]	eclampsia	-	yes	yes	-	midwife	no
4	yes	4	male	1900	-	-	-	-	yes	-	-	midwife	no
5	yes	4	male	2900	-	-		-	-	-	yes	obstetrician	yes
6	no	2	female	980	-	yes[3]	no fetus heartbeat	-	-	-	-	midwife	no
7	yes	3	female	1750	-	-	-	-	-	-	yes	obstetrician	yes
8	yes	4	male	3300	-	-	-	-	yes	-	-	midwife	no
9	yes	3	male	1600	-	-	-	yes	-	-	-	midwife	no
10	yes	4	male	3000	-	-	-	yes	-	-	-	obstetrician	yes
11	yes	4	female	1900	-	-	-	-	-	yes	-	midwife	no
12	no	4	male	3100	yes*	yes[1]	no fetus heartbeat	yes	yes	-	-	midwife	no
13	yes	4	male	2000	-	-	-	-	yes	-		midwife	no
14	yes	4	male	3500	-	-	-	-	-	-	--	midwife	yes
15	yes	1	male	1200	-	-	-	yes	yes	-	-	midwife	yes
16	yes	4	female	3990	-	-	-	-	yes	-	-	midwife	N0

This is the Table 1 legend.

*Malformation in two stillbirths: Multiple malformations in the rib cage, spina bifida, clubfoot and hydrocephalus

[1] Caue district, [2] Cantagalo, [3] Lobata

[ǂ]sub-categories of preterm birth, based on gestational age: 1=extremely preterm (less than 28 weeks); 2=very preterm (28 to 31 weeks); 3=moderate to late preterm (32 to 36 weeks); 4=term newborns (37 to 41) and 5= posterm newborn (>42)
