# Supplementary material for: Factors associated with perinatal and neonatal deaths in Sao Tome & Principe: a prospective cohort study
Source: Front Pediatr. 2024 Feb 16;12:1335926. doi: 10.3389/fped.2024.1335926 (PMC10904650; doi:10.3389/fped.2024.1335926)
Supplement: Supplementary file 2 [file Datasheet2.pdf]

Additional file 2 Death-outcome group: characteristics of the six live births with a neonatal death (n=6)

Neonatal death	Time of death (range)	Main cause of death	Sex	Birth weight	Prematurity	Major malformation	Birth asphyxia	NCU admission	PROM	Meconium	Eclampsia	Birth attendant	Dystocia
ǂENND 1	< 24h	Prematurity-related death	male	1230	yes	no	no	yes	yes	yes	no	home delivery	no
ENND 2	< 24 h	Major malformation + Birth asphyxia	male	3000	no	yes*	yes	yes	no	no	no	obstetrician	yes
ENND 3	Day 1-7	Sepsis + Birth asphyxia	male	2200	yes	no	yes	yes	no	yes	yes	midwife	no
ENND 4	Day 1-7	Major malformation + Birth asphyxia	female	3450	no	yes*	yes	yes	no	yes	no	midwife	no
LNND 5	Day 8-10	Sepsis + Birth asphyxia + Brachial plexus lesion	male	4250	no	no	yes	yes	no	yes	no	midwife	no
LNND 6	Day 10-15	Probably late sepsis	female	3800	no	no	no	no	no	no	no	obstetrician	yes

This is the Table 2 legend.

Abbreviations: ANC – antenatal care; NCU – Neonate Care Unit; PROM – prolonged rupture of membranes; yo – years old; ENND – early neonatal death.

*Major malformations identified ENND 2: hydrocephaly and ENND 4: thoracic malformation

ǂMaternal characteristics: ENND 1 (mother age range 35-40 years old, G7 P6, 1 ANC visit); ENND 2 (mother age range 15-19 years old, G0 P0, 6 ANC visits); ENND 3 (mother age range 20-24 years old, G1 P0, 4 ANC visits); ENND 4 (mother age range 35-40 years old, G6 P5, 4 ANC visits); LNND 1 (mother age range 35-40 years old, G5 P3, 8 ANC visits); LNND 2 (mother age range 35-40 years old, G2 P1, 6 ANC visits).
